# smyRNA: A Novel Ab Initio ncRNA Gene Finder

**DOI:** 10.1371/journal.pone.0005433

**Published:** 2009-05-05

**Authors:** Raheleh Salari, Cagri Aksay, Emre Karakoc, Peter J. Unrau, Iman Hajirasouliha, S. Cenk Sahinalp

**Affiliations:** 1 School of Computing Science, Simon Fraser University, Burnaby, British Columbia, Canada; 2 School of Computer Science, University of Waterloo, Waterloo, Ontario, Canada; 3 Department of Molecular Biology and Biochemistry, Simon Fraser University, Burnaby, British Columbia, Canada; Lehigh University, United States of America

## Abstract

**Background:**

Non-coding RNAs (ncRNAs) have important functional roles in the cell: for example, they regulate gene expression by means of establishing stable joint structures with target mRNAs via complementary sequence motifs. Sequence motifs are also important determinants of the structure of ncRNAs. Although ncRNAs are abundant, discovering novel ncRNAs on genome sequences has proven to be a hard task; in particular past attempts for ab initio ncRNA search mostly failed with the exception of tools that can identify micro RNAs.

**Methodology/Principal Findings:**

We present a very general ab initio ncRNA gene finder that exploits differential distributions of sequence motifs between ncRNAs and background genome sequences.

**Conclusions/Significance:**

Our method, once trained on a set of ncRNAs from a given species, can be applied to a genome sequences of other organisms to find not only ncRNAs homologous to those in the training set but also others that potentially belong to novel (and perhaps unknown) ncRNA families. Availability: http://compbio.cs.sfu.ca/taverna/smyrna

## Introduction

A non-coding (nc)RNA is any RNA that is transcribed but not translated into a protein. ncRNAs have diverse functionalities in the cell such as regulation of gene expression by means of interacting with target mRNAs and prohibiting their translation [1]. Recent discoveries have pointed to the abundance of ncRNAs; for example up to 62% of the mouse genome sequence seems to be transcribed but not translated [2], [3]. Determining the sequence, structure and functionality of ncRNAs provides a major scientific challenge that will need to be addressed in the coming years.

The advent of novel sequencing technologies (such as the pyrosequencing based technology developed by 454 Life Sciences [4]) promises a significant growth in the number of available ncRNA sequences. Unfortunately, the size limitations on the fragments that can be sequenced by these technologies necessitate the development of alternative, in particular computational approaches to the exploration of longer ncRNAs.

Discovering ncRNA genes on a given genome sequence is a challenging task, quite different from that of discovering protein coding genes. Unlike protein coding RNAs, ncRNAs lack key sequence signals such as start and stop codons, codon bias or promoter regions [5]. Furthermore, protein coding RNAs are resistant to frame shifts and they have many more silent mutations than other parts of genome; such observations which can be used towards the discovery of protein coding genes are not valid for ncRNA genes. Thus existing computational models and tools for protein coding gene discovery can not be applied directly to ncRNA genes discovery; novel approaches are needed.

In [5] three main computational problems related to the exploration of ncRNAs are identified: (i) ncRNA validation: given one or more input sequences, determine whether they are ncRNAs or not; (ii) ncRNA homolog search: given one or more members of an ncRNA family (with or without structural information), search for other members of the same family on a geneome sequence; (iii) ab initio ncRNA discovery: given general sequence and structural properties of ncRNAs discover novel ncRNA sequences (which may not belong to any known ncRNA families) in a genome sequence. Following [5] we overview known approaches to each one of these problems, discuss the main challenges and finally summarize our contributions.

### ncRNA validation

Arguably the most successful set of computational tools for ncRNA exploration have been developed for the problem of ncRNA validation. Many of these tools aim to detect conserved structures among functionally similar ncRNAs of related species. For example,the QRNA program by Rivas and Eddy [6] looks at covariation patterns in an alignment of two sequences and decides whether they are ncRNAs, protein coding RNAs or neither. The probability of an ncRNA is calculated through the distribution of covarying mutations (and the structure they imply) modeled via a a stochastic context free grammar. Coding region probability is calculated using a *Hidden Markov Model (HMM)* which considers mutations that do not change the final protein product. The probability of the sequences being neither an ncRNA nor a protein coding RNA is calculated by another HMM which considers mutations that take place independent of position. The final classification is performed by a Bayes classifier.

A follow-up to QRNA by Bernardo et al. is ddbRNA [7], a program for finding all potential stems that are conserved in an alignment of multiple sequences. The program commits itself to a particular stem according to its composition especially with respect to the number of covarying mutations in it. If the number and distribution of such stems are significantly different from those that can occur in the randomly shuffled version of the alignment, then the alignment and thus the sequences are declared as ncRNAs.

Another paper in this direction by Coventry et al. presents a similar tool, MSARI [8] for identifying conserved stems in an alignment of a number of (10–15) potential ncRNA sequences. In contrast to ddbRNA, MSARI uses the RNAfold program for predicting the independent secondary structures of the input sequences. For each predicted stem MSARI considers all 7 nt blocks which are aligned to each other (to produce potential common stem loops); each such alignment is considered to be of interest as a function of its composition, especially the number of covarying mutations it involves. A distribution mixture model is then used to determine if the resulting set of stem loops are significant.

The program EvoFold [9] by Pedersen et al. extends QRNA - which works on a pair of sequences- to multiple sequences by a phylogenetic footprinting approach. The paper [9] constructs a whole-genome alignment of the human with 7 other species (with varying evolutionary distances to human), to obtain over 1 million conserved regions covering 3.7% of the whole human genome. From these regions, EvoFold was able to return over 48000 candidate conserved structures; approximately 18500 of these structures are estimated to be true positives, forming about 10000 ncRNA transcripts.

Recently, Washietl et al. developed the program RNAz [10] which incorporates structural conservation with thermodynamic stability. RNAz uses up to 6 sequences from various species to decide if the sequences are indeed ncRNAs from the same family. A secondary structure for each input sequence is predicted via RNAfold and a consensus structure is obtained by aligning the sequences using alifold [11]. A structure conservation index (SCI), a measure of secondary structure conservation among input sequences, is derived from these two outputs. A z-score for each sequence is calculated with the help of SVM regression, which gives a statistical quantification for the thermodynamic stability of the input sequences compared to random sequences. Another SVM is then used to classify the input as ncRNA or not.

### ncRNA homolog search

Existing tools for detecting members of a known ncRNA family in a genome sequence typically use covariance models (CM), probabilistic models that can describe a family of RNA secondary structures. In the context of CMs an RNA secondary structure is represented as an ordered tree where nodes are states representing base pairs, single nucleotides, insertions or deletions. Each state has symbol emission probabilities which correspond to probabilities of observing each nucleotide (or base pair) at that state and transmission probabilities which correspond to probabilities of switching from the current state to a following state or not switching at all. Special bifurcation, begin and end states are used for defining the tree structure itself. The model parameters and tree structure is trained with members of an ncRNA family. The CM can then be aligned to a given sequence to determine homologs.

CMs were first introduced by Eddy and Durbin in 1994 [12]. Since then, various methods have been proposed which take advantage of CMs in ncRNA discovery. One such example is the INFERNAL package [13] which is used for annotation in the Rfam database [14] that includes over 500 ncRNA families coded by more than 13000 ncRNA genes. A small set of representative known ncRNA sequences are annotated in seed alignments by human curators with secondary structure information for each ncRNA family. The remainders of the Rfam ncRNAs were annotated using the INFERNAL package.

### Ab initio ncRNA discovery

As mentioned earlier this paper focuses on the problem of ab initio ncRNA discovery, which, given a genome sequence and a set of ncRNAs asks to discover novel ncRNAs that may or may not belong to known ncRNA families. As a recent survey on RNA gene prediction [5] states ab initio gene prediction is the most challenging case of RNA gene prediction: “In the general case ab initio RNA gene prediction is still a more or less unsolved problem”.

The only general approach for ab initio ncRNA discovery so far is based on thermodynamic stability. NCRNASCAN program, developed by Rivas and Eddy [15] aims to use structural stability as an indicator of ncRNA presence. The program employs three different models for assessing the structural stability, each of which can be used to scan the input genome sequence towards identifying stable structures. Unfortunately, because ncRNAs in general are not significantly more stable than random genome sequences the applicability of NCRNASCAN is very limited. The only ncRNA family for which this approach has been reported to attain success is micro (mi)RNAs, which indeed have significantly more stable structures in comparison to random sequences [16]. The RNALfold program by Hofacker et al. [17] can in fact effectively discover miRNAs by detecting short locally stable structures in a genome sequence. Given any number of evolutionary related RNA genes SimulFold [18] predicts novel RNA genes based on the evolutionarily conserved RNA structure rather than the thermodynamic or MFE structure.

Carter et al. [19], by using a machine learning approach based on differences in compositional and structural parameters present in known RNAs compared to non-coding sequences, achieved an improvement in RNA genes identification in bacterial and archaeal genomes.

In a recent approach [20], which does not require a multiple-sequence alignment as input, RNA motif prediction with RNA homolog search has been integrated. This approach was able to improve the quality of the RNA motifs discovery in prokaryotes.

It is tempting to apply some of the available techniques on ncRNA validation and ncRNA homolog search to the problem of ab initio ncRNA discovery. Unfortuantely: (1) ncRNA validation techniques, in general, rely heavily on the availability of some good initial sequence alignment between two or more potential ncRNAs. However, homologous ncRNAs typically have poor sequence conservation which considerably limits the applicability of conserved region detection towards discovery of species specific ncRNAs [5], [21]. (2) In addition, the number of conserved regions in a given genome is far too large for a genome-wide scan. Thus, conserved structure search methods make use of readily available data sets: RNAz makes use of conserved structure databases covering only a small portion of the genome [10], MSARI needs alignments of conserved sequences from as much as 10 different species [8] and EvoFold needs a costly genome-wide alignment of several genome sequences [9]. (3) ncRNA homolog search methods, especially those employing covariance models, clearly aim to discover members of known ncRNA families and are not applicable to ab initio ncRNA discovery.

## Methods

smyRNA (structural sequence motifs yielding to ncRNAs) is a simple ab initio ncRNA discovery tool which is based on the premise that certain sequence motifs act as important determinants of ncRNA structures and have differential distribution among ncRNAs and the background genome sequences.

Given a genome sequence *G*, let *G*[j] denote the *j* ^th^ nucleotide of *G*, and let *G*[i, j] denote the substring including *G*[i]…*G*[j]. Now denote by *N*, the set of all ncRNAs known in *G* and by 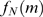 the total number of occurrences of a *k*-mer motif *m* (for a specific value of *k*) among all ncRNA sequences in *N*. Similarly let 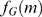 be the total number of occurrences of *m* in *G*. Thus the frequency of *m* in *N* and *G* can respectively be defined as
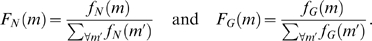



The log-likelihood ratio for *m* residing in an ncRNA is thus 
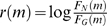
. A positive value for 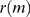 implies the motif is more frequent in ncRNAs, and a negative implies the contrary. Thus, the log-likelihood score of a substring *S* being (a part of) an ncRNA, in terms of the frequencies of all *k*-mer motifs it includes can be defined as 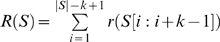 where *S*[i : i+k−1] is the *k*-mer sequence motif starting at position *i* in subsequence *S*.

Given *G* and *r* the following dynamic programming formulation can identify those substrings *S* for which *R*(*S*), the log-likelihood score, is maximum possible; i.e., it is not possible to increase *R*(*S*) by extending such an *S* in any direction.
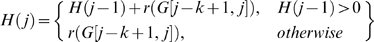
Here *H*(*j*) denotes the maximum possible *R*(*G*[i, j]) among all substrings *G*[i, j] for which i≤j. Let 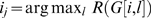, i.e., the value of *i* which maximizes the log-likelihood score for *G*[*i*, *j*]. We leave it to the reader to observe that for any location *h* on *G*, if 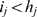 then 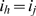. Thus for a given location 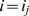 on *G*, one can define 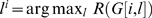, i.e. the location for which *R*(*G*[*i*,*l*]) is maximized. Consider those locations *i* for which 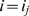 for some *j*, and then for each such *i* consider the corresponding location 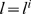. The substring *G*[*i*,*l*] will have the property that no substring 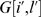 which overlap with *R*(*G*[*i*,*l*]) can have 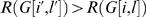; thus 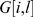 will be a maximally scoring substring. Once the above values of *H*(*j*) are obtained, it is possible to obtain all maximally scoring substrings of *G* in linear time through a simple greedy algorithm.

For training, given a genome sequence *G* and a set of ncRNAs N, smyRNA processes G and all sequences in N to calculate F_G(m) and F_N(m) for all k-mer motifs *m*. Then *r(m) = log(F_N(m)/F_G(m))* is calculated for each k-mer motif *m*. Note that the number of all possible k-mer motifs for the 4 letter DNA alphabet is *4^k^*. Thus the time and memory requirement for training is *O(|G|+4^k^)* which is linear in length of G for small values of k.

Based on the trained log-likelihood ratio *r*, smyRNA can locate other ncRNAs on an input genome sequence *G* by determining the maximally scoring substrings of the input sequence *G*. Those substrings whose score is over a user defined threshold *t* are then declared as ncRNA candidates.

## Results

For assessing the predictive power of smyRNA, we applied the following testing strategy: (1) We trained smyRNA on a given genome sequence and its collection of known ncRNAs and then tested it on another genome sequence. To ensure that the genome sequences include no unknown ncRNA genes we randomly shuffled the bases on the background sequence while leaving the known ncRNA genes intact. More precisely, we used the following shuffling algorithm for genome sequence G:

1. Remove all known ncRNA genes from genome sequence.
2. Generate a large random integer *a* (*a>c.|G|*, *c* is *a* user defined constant).
3. For *a* times repeat steps 4–5.
4. Generate two random integers *i,j*.
5. Swap G[*i*] and G[*j*].
6. Insert each ncRNA gene at some random position.

Once the PPV (positive predictive value) and sensitivity for varying threshold values are determined, the “best possible” threshold value (providing a good tradeoff between the PPV and sensitivity) is selected which is applied to discover novel potential ncRNA genes later. (2) We also applied leave-one-out cross validation experiments to a set of known ncRNAs of a genome. In each iteration we removed one of the known ncRNAs from the collection and trained smyRNA on the shuffled genome sequence and remaining set of ncRNAs. We then applied smyRNA to the unshuffled genome sequence and measured the PPV and sensitivity. (3) Finally we applied smyRNA to the unshuffled genome sequences. We verified the candidates for unknown ncRNA genes through the use of existing ncRNA validation techniques.

In our tests, we primarily used the E.coli K12 genome sequence, perhaps the best studied organism with respect to ncRNAs. Rfam database v8.1 [14] presents 164 ncRNAs from 63 different families for E.coli K12. For cross validation purposes, we used the S.flexneri 2a str. 301 a bacteria highly divergent from E.coli, which has a rich set of ncRNA sequences in Rfam database v8.1 [14]. In Rfam database there are 183 ncRNAs from 64 families for S.flexneri 2a str. 301.

Our first experiment aimed to (re)discover all known ncRNA genes in the S.flexneri genome via smyRNA - which was trained by the use of the complete set of known E.coli ncRNAs and the E.coli genome. Note that we excluded all tRNAs from the training set. As mentioned above, we randomly exchanged pairs of nucleotides (exchanging each nucleotide at least once) in both the training genome sequence (E.coli) and the test genome sequence (S.flexneri) while retaining the known ncRNA genes intact in both sequences.

**Figure 1:**
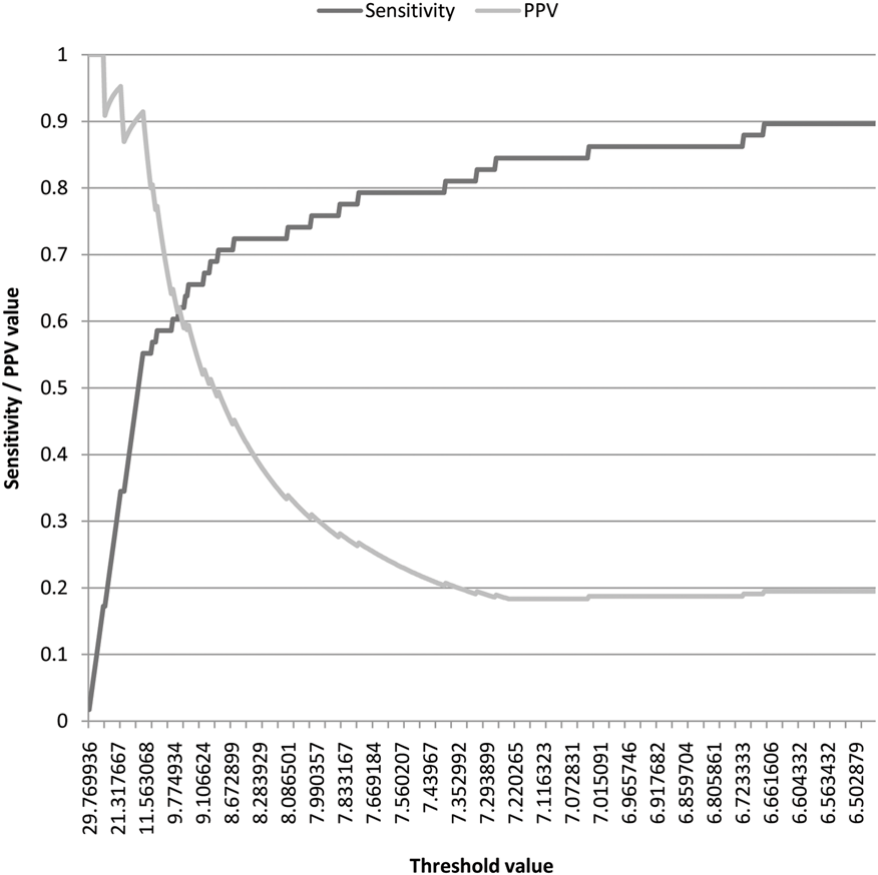
Specificity and sensitivity values for different thresholds of smyRNA trained on E.coli and tested on S.flexneri based on the highest 1,000 ranking predictions.

For different values of k, we determined both the PPV (tp/(tp+fp) where tp and fp are the number of true and false positives respectively) and the sensitivity (tp/(tp+fn) where f n is the number of false negatives) of smyRNA for all possible threshold values; the results are shown for k = 5 (which gave the best results) in Figure 1. As can be seen, a (log-likelihood score) threshold value of 9.6 gives a PPV and sensitivity of 0.61 providing a reasonable trade-off between the two measures. To obtain a “more meaningful” PPV of 0.80, one has to increase the (log-likelihood score) threshold value to 11 for which sensitivity drops only slightly to 0.58. As a result we use the log-likelihood cutoff of 11 to determine putative ncRNA sequence in the next set of experiments.

**Figure 2:**
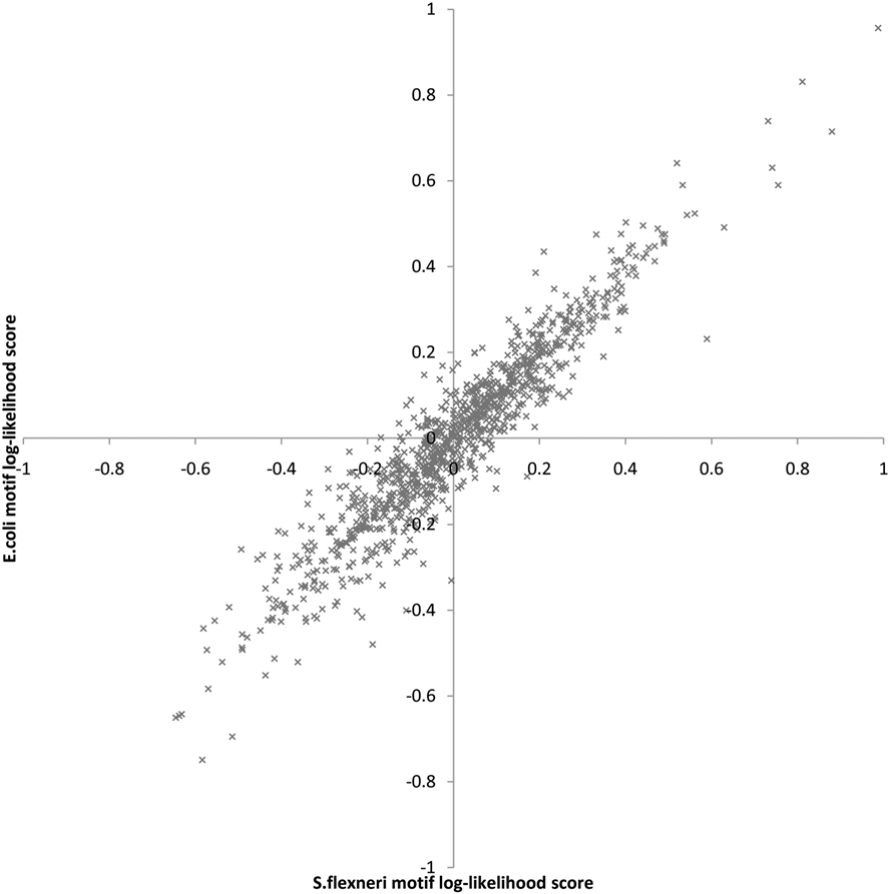
Log-likelihood score comparison of pentamer log likelihood scores from *S. flexneri* and *E. coli*. Each data point corresponds to a pentamer p positioned at (x, y) where x is the log likelihood score of p in *E. coli* and y in *S. flexneri*.

Perhaps it is not surprising that the E.coli trained smyRNA achieved high PPV and reasonable sensitivity on (permuted) S.flexneri as 1024 possible pentamer motifs from the 4 letter DNA alphabet have very similar log-likelihood scores in these two species. Figure 2 depicts the comparative log-likelihood scores of all possible pentamer motifs for the genome sequences and the known ncRNAs of the two species. Notice that the distribution does not deviate significantly from the x = y line, which corresponds to perfect match between the log-likelihood scores of pentamers in E.coli and S.flexneri. This distribution can not be observed for the other values of k. Even k = 4 results in different distribution.

In a separate experiment, we applied smyRNA to a set of genomes with different types. We select genomes that have reasonably short genome sequences and rich sets of ncRNA sequences in Rfam database. We used a cutoff log-likelihood score of 11 for the E.coli trained smyRNA. Table 1 shows the number and percentage of discovered ncRNA genes by smyRNA on different genomes. Note that unlike S.flexneri, the distribution of the log-likelihood scores of pentamer motifs in these genomes does not show a high similarity to that in E.coli. These genomes (especially those from Eukaryotic species) are distinct from E.coli, which has been used for training purposes, and smyRNA still achieves high predictive power on rediscovering known ncRNAs. More specifically about 74% of known ncRNAs were discovered by smyRNA. Several of these ncRNAs belong to ncRNA families which are not present in E.coli.

**Table 1:** Predictive power of smyRNA on different genomes.

Genome	Type	Length(nt)	# of known ncRNAs	# of known ncRNAs returned by smyRNA	# of all subsequences returned by smyRNA
Cyanophora paradoxa cyanelle	Eukaryota	135,599	40	36(90%)	61
Kluyveromyces lactis strain NRRL Y-1140 chromosome B of strain NRRL Y-1140 of Kluyveromyces lactis	Eukaryota	1,320,834	32	25(78%)	104
Yarrowia lipolytica chromosome A of strain CLIB122 of Yarrowia lipolytica	Eukaryota	2,303,261	86	71(83%)	102
Yersinia pestis strain CO92	Bacteria	4,653,728	118	83(70%)	140
Salmonella enterica subsp. enterica serovar Choleraesuis str. SC-B67	Bacteria	4,755,700	159	109(69%)	141
Vibrio cholerae O1 biovar eltor str. N16961 chromosome I	Bacteria	2,961,149	126	101(80%)	217
Shigella flexneri 2a str. 301	Bacteria	4,607,203	183	120(66%)	254

*E. coli* has been used for training and threshold score is set to t = 11. Number and percentage of discovered ncRNAs (presented in fifth column) shows the accuracy of smyRNA in predicting ncRNA genes on different genomes.

Next we performed leave-one-out cross validation experiments. We trained smyRNA on a set of known ncRNAs and shuffled genome sequence of E.coli, and tested it on unshuffled genome sequence of E.coli. Among the complete set of all ncRNAs 65% of them were discovered by smyRNA. To make sure that smyRNA is not simply performing homology search, we repeat the experiment by using one ncRNAs from each family. In this case smyRNA discovered 42% of ncRNA sequences.

As mentioned earlier, the final experiment we performed aimed to find novel ncRNAs in unshuffled genome sequences. We trained smyRNA on the set of known E.coli ncRNA sequences as well as the E.coli genome sequence. Then we tested smyRNA on E.coli genome to determine both known and possibly unknown ncRNA sequences. As per above, we determined the putative ncRNA sequences whose log-likelihood score was at least 11; so that 34 out of 76 known ncRNAs (with the exception of tRNAs and rRNAs) and 191 previously unknown ncRNA candidates were identified. Among the unknown ncRNA candidates, 81 have overlaps with ORFs. On average their GC content is 41.4% and their length is 615 nt. To make sure that they are not coding RNAs, we picked a random sample of them and BLASTed them to see whether they have hits to coding RNA. None of these referred a hit.

We applied RNAz v1.0 program, perhaps the best known ncRNA validation tool, which is known to achieve significantly higher specificity/sensitivity values than competitive methods [22] to validate each such candidate as follows. We searched each candidate sequence in the complete genomic BLAST databases made available by NCBI. We used nucleotide collection database, and identified up to 5 highly conserved sequences (RNAz requires at least 2 and can take into account at most 6 sequences) with conserved length greater than 20 nt, and E-value<10. (The very fact that each of these candidates was highly conserved, yet not perfectly conserved, in other species suggests functionality.) The candidate ncRNA and its BLAST hits (which typically were from other species with average Evalue less than 0.01) were first aligned via the ClustalW v1.83 program [23] and then fed to the RNAz v1.0 program for validation. Among the 191 candidates, 98 were classified as ncRNAs by RNAz. Among them, 35 have overlaps with ORFs. Also on average their GC content is 41.7% and their length is 566 nt. The average returned “structure conservation index (SCI)” by RNAz for all of the 191 candidates was 94% and for those classified as ncRNA was 95%.

Note that Washietl et al., the developers of the RNAz program were able to identify only 89 putative ncRNAs (fewer than what smyRNA was able to find on the E.coli genome) in their study which was based on the CORG database that includes 4263 (annotated) conserved non-coding regions from 5 species (human,mouse, rat, Fugu and zebrafish) [24].

## Discussion

This paper presents a novel ab initio ncRNA gene discovery tool which exploits differential distribution of k-mer motifs (in particular pentamer motifs) among ncRNAs with respect to the background genome sequence. Based on the k-mer motif distribution we showed how to compute log-likelihood scores for a specific sequence to be in a potential ncNRA sequence and how to identify maximally scoring subsequences of a genome which can then be considered as a candidate ncRNA gene. We showed how to train the resulting tool via a given set of ncRNAs and the background genome sequence towards identifying the most likely set of ncRNA genes in a test sequence. We trained our tool, which we call smyRNA, on the complete set of E.coli ncRNAs and applied it to the S.flexneri genome sequence on which was randomly shuffled with the exception of known ncRNA genes. smyRNA was able to identify a significant fraction of known S.flexneri ncRNAs while returning only a small number of false positives. We then applied the E.coli trained smyRNA again on the unshuffled E.coli sequence towards identifying unknown ncRNA genes. The 191 top ranking ncRNA candidates were then verified by the RNAz program. Among them, RNAz classified 98 of them (more than half) as ncRNAs. Thus we conclude that smyRNA provides a simple, efficient and potentially powerful approach to ab initio ncRNA gene discovery problem.
